# Serially coupling hydrophobic interaction and reversed-phase chromatography with simultaneous gradients provides greater coverage of the metabolome

**DOI:** 10.1007/s11306-014-0770-7

**Published:** 2015-01-11

**Authors:** Jennifer Haggarty, Madalina Oppermann, Matthew J. Dalby, Richard J. Burchmore, Ken Cook, Stefan Weidt, Karl E. V. Burgess

**Affiliations:** 1 Polyomics, University of Glasgow, 211 Wolfson Wohl Translational Cancer Research Centre, Garscube Campus, Glasgow, G61 1QH UK; 2 Thermo Fisher Scientific, Hemel Hempstead, UK; 3 Centre for Cell Engineering, University of Glasgow, Glasgow, UK

**Keywords:** HILIC, Reversed phase, LC–MS, Organic acids, Bile acids, Beer

## Abstract

The serial coupling of a reversed-phase liquid chromatography (RPLC) column to a hydrophilic interaction liquid chromatography (HILIC) column has been developed in recent years for the detection of polar and nonpolar metabolites. TCA intermediates, bile acid standards and numerous polar and non-polar metabolites extracted from beer were analysed using a combined RPLC/HILIC method. Non-polar metabolites were retained by the RPLC column. Polar metabolites not retained by the RPLC column were retained and separated by the HILIC column. The results from this study validate this simple yet powerful metabolomics approach.

**Electronic supplementary material:**

The online version of this article (doi:10.1007/s11306-014-0770-7) contains supplementary material, which is available to authorized users.

## Introduction

The field of metabolomics has the difficult task of attempting to characterise and quantifying the vast array of metabolic compounds present in biological systems. The diversity in these compounds creates analytical problems. Chromatographic techniques tend to be effective for specific compound classes. For example, reversed phase (RP) HPLC coupled to mass spectrometry, has been used very successfully to cover a wide range of non-polar compounds; however, polar and ionic compounds do not retain well on these columns without the use of ion pairing reagents—and so give little to no separation. Some of the most important compound classes in metabolomics fall into these groups: carbohydrates, organic acids, amino acids and nucleotides chromatograph poorly on RP HPLC, but retain and separate well with some hydrophilic interaction liquid chromatography (HILIC) columns, more specifically—the ZIC-pHILIC zwitterionic surfactant column. Conventionally, analysis of the polar and non-polar metabolites in a sample requires two separate chromatographic runs. This complicates sample preparation due to the requirement for the sample buffer to be appropriate for the solvent system used (Hemström and Irgum 2006).

Two chromatographic methods are available to improve the retention of analytes. The first is two-dimensional chromatography, in which samples are run on one column and the resulting fractions are subsequently analysed using a column with a different stationary phase. This method is used often in proteomics, e.g. using strong cation exchange (SCX) followed by RP (Edelmann 2011). RPLC/HILIC systems have been utilized in many research areas, including metabolomics (Di Palma et al. 2011; Gilar et al. 2005; Kalili and de Villiers 2010; Wang and Lehmann 2008; Lam et al. 2010). Although the solvents used for RPLC and HILIC are the same, the need for diametrically opposed organic concentration at the start of each separation has led to the development of on-line RPLC/HILIC valve switching systems (Wang et al. 2008; Thomas et al. 2010; Wang and Lehmann 2008). These methods, although successful, are not suited for routine use in most laboratories as dedicated interfaces are required for handling mobile phase incompatibilities. The need to run samples on two columns consecutively also leads to increased analysis times and difficulty of data analysis. A simpler approach, coupling two columns together and running the gradients simultaneously is less routinely described. This method has been reported for the separation of polar and nonpolar pharmaceuticals (Louw et al. 2008) and phenols (Greco et al. 2013) by serially coupling a C18 column with a HILIC column.

This paper describes a metabolomics method in which a sample can be injected onto both RP and HILIC columns in tandem for the analysis of polar and non-polar compounds in a single injection. This method allows for the independent optimisation of both columns and removes the need for separate sample preparations or the addition of ion pairing reagents.

The practicality and the reliability of the method were investigated by the analysis of TCA intermediates and bile acid standards. Subsequently, the feasibility of the coupled method for non-targeted analysis of large nonpolar compounds and small highly polar compounds in a complex sample (beer) was evaluated.

## Experimental

### Chemicals and reagents

HPLC gradient grade water was purchased from VWR (Chicago, USA). Absolute ethanol and acetonitrile were purchased from Fisher Scientific (Leicestershire, UK). Ammonium carbonate, deoxycholic acid, cholic acid, dehydrocholic acid, chenodeoxycholic acid, DL-isocitric acid trisodium salt hydrate, cis-aconitic acid, α-ketoglutaric acid disodium salt hydrate, sodium succinate dibasic, sodium fumarate dibasic, DL-malic acid and sodium pyruvate were all purchased from Sigma-Aldrich (Bornem, Belgium). All chemicals were of analytical-reagent grade.

### Standards

All of the standards were prepared to a concentration of 10 µM in water. Stock standard solutions of deoxycholic acid, cholic acid, dehydrocholic acid and chenodeoxycholic acid were prepared in 100 % hot ethanol. The standards were then diluted at a 1:1 ratio with H_2_O and then diluted 1:100 in 100 % H_2_O, to a final concentration of 10 µM. (The structures of these compounds can be found in Fig. 1 in Supplementary Information).

10 µL of each sample was injected onto the column in quadruplicate.

### Beer samples

Samples of a bitter style branded ale were extracted at a ratio of 1:3:1 chloroform:methanol:beer. 250 µL samples were dried using N_2_ and resuspended in 200 µL of 10 % acetonitrile in water.

10 µL of each sample was injected onto the column in octuplicate.

To evaluate the long term reproducibility of the method, analysis of the combined method was carried out in three batches over 5 days, while the individual column analyses were done over a single day.

### Identification

Organic acid and bile acids were matched to authentic pure standards by retention time and accurate mass and can therefore be classified under the alphanumeric metabolite coding scheme as HRM${\text{S}}^{1}_{\text{a}}$, R_ta_ as described in Sumner et al. (2014). Beer metabolites were matched to compounds putatively by accurate mass or accurate mass and fragment spectrum match to the MassBank library (Horai et al. 2010) and may be classified as HRMS^1^ or ${\text{HRMS}}^{1} {\text{MS}}^{2}_{\text{PL}}$ respectively (Sumner et al. 2014).

### Instrumentation

A Thermo Scientific Ultimate 3000 RSLC system (Thermo Scientific, CA, USA) was used. Column temperature was maintained at 25 °C. The system was coupled with a Thermo Scientific Exactive Orbitrap system equipped with a HESI II interface (Thermo Scientific, Hemel Hempstead, UK). Acquisition was carried out in positive and negative switching mode. The capillary temperature was 275 °C with a data acquisition mass range of 70–1400 *m/z*. Thermo Xcalibur™ (version 2.2.42) was used for instrument control and data acquisition. Fragmentation for annotation of beer metabolites was performed on an identically configured Thermo Scientific Q-Exactive with a normalised collision energy of 50 and isolation width of 1 Da.

**Fig. 1:**
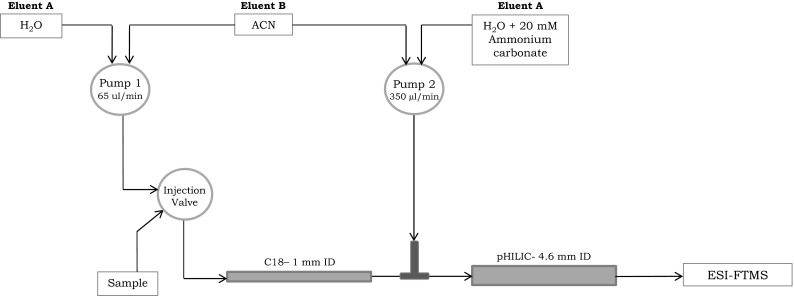
Schematic diagram of the RPLC/pHILIC/ESI-FTMS

For the first separation a Hypersil GOLD (100 × 1.0 mm, 1.9 µm) (Thermo Scientific, Hemel Hempstead, UK) was used and the second separation was performed with a SeQuant^®^ ZIC^®^-pHILIC column (150 × 4.6 mm, 5 µm) (Merck KGaA, Darmstadt, Germany). The two columns were coupled in series through a T-piece. The third port of the T-piece was connected to the second pump. A scheme of the instrumental set-up is given in Fig. 1. All metabolites were detected in negative ionisation mode except where stated.

### Chromatographic conditions

The RPLC mobile phase was a combination of LC–MS-grade water (solvent A) and acetonitrile (ACN) (solvent B). The HILIC mobile phase was a mixture of LC–MS-grade water + 20 mM ammonium carbonate, pH 9, (solvent A), ACN (solvent B) and the solvent from the RP column. The following RP gradient was applied at a rate of 65 µL/min: a concentration of 5 %B was held for 2 min and then increased to 95 % B over 15 min, where it was held for 5 min, followed by equilibration at 5 % B, held for 12 min. The HILIC gradient was applied at a rate of 350 µL/min: a 90 % B was held for 5 min and then decreased to 20 % B in 15 min and then decreased to 5 % B and held for 5 min, followed by reconstitution of the starting conditions within 0.1 min and re-equilibration with 90 % B for 12 min. This resulted in a total analysis time of 37 min. (Table 1, Supplementary Information).

### Evaluation of reproducibility

Retention times were obtained for each peak using the Quan Browser software, part of the Thermo Xcalibur™ (version 2.2.42) suite. Retention times were averaged and the standard deviation was calculated. Relative standard deviations were obtained for each compound and expressed as percentages.

## Results & discussion

### Standards

**Fig. 2:**
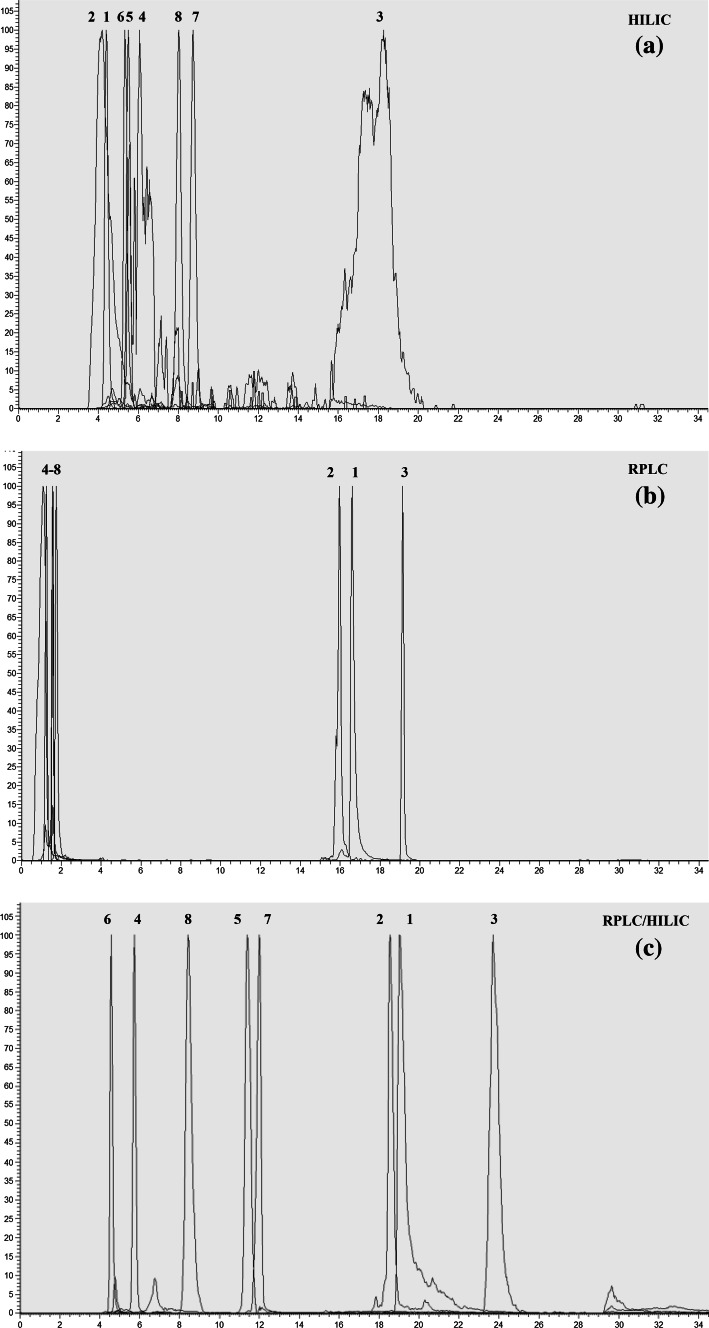
EICs of selected beer metabolites with annotations. (*1*) ad-/Humulone, (*2*) iso-/Xanthohumol, (*3*) cohumulone, (*4*) syringaldehyde, (*5*) tyrosine (*6*) ethyl vanillin, (*7*) succinic acid, (*8*) phenylalanine. Injection volume 10 µL. Detection: ESI-FTMS. Separation by **a** HILIC, **b** RPLC and **c** RPLC/HILIC

The polar organic acids that were retained on the pHILIC column were not retained when using the RPLC column alone (Fig. 2a, b, Supplementary Information). In contrast to the RPLC, when the compounds were analysed using the RPLC/HILIC method, all of the organic acids were retained (Fig. 2c, Supplementary Information). The RPLC/HILIC method showed good retention time reproducibility, with RSD values for each compound under 5 % (Table 1).

**Table 1:** Average retention times (RT) and RSDs of selected organic and bile acids

						HILIC		RPLC		RPLC/HILIC	
Metabolite	Elemental formula	KEGG ID		Metabolite ID code^a^	[M−H]	Average RT (min)	% RSD	Average RT (min)	% RSD	Average RT (min)	% RSD
Isocitric acid	C6H8O7		C00311	HRM${\text{S}}^{1}_{\text{a}}$, R_ta_	191.01973	14.72	0.34869	0.88	3.93648	15.33	0.219251
cis-Aconitic acid	C6H6O6		C00417	HRM${\text{S}}^{1}_{\text{a}}$, R_ta_	173.00916	13.14	0.39043	0.85	1.38564	14.27	0.499636
2-Oxoglutaric acid	C5H6O5		C00026	HRM${\text{S}}^{1}_{\text{a}}$, R_ta_	145.01425	9.46	0.61052	0.80	0.71869	11.88	1.018596
Succinic acid	C4H6O4		C00042	HRM${\text{S}}^{1}_{\text{a}}$, R_ta_	117.01933	9.08	0.71683	0.85	0.70696	11.71	0.861405
Fumaric acid	C4H4O4		C00122	HRM${\text{S}}^{1}_{\text{a}}$, R_ta_	115.00368	10.64	1.26948	0.80	1.25000	12.37	0.652205
Malic acid	C4H6O5		C00149	HRM${\text{S}}^{1}_{\text{a}}$, R_ta_	133.01425	10.64	0.98752	0.83	0.70124	12.60	0.577116
Pyruvic acid	C3H4O3		C00022	HRM${\text{S}}^{1}_{\text{a}}$, R_ta_	87.00877	5.03	0.80400	0.84	4.48926	5.06	1.418423
Deoxycholic acid	C24H40O4		C02528	HRM${\text{S}}^{1}_{\text{a}}$, R_ta_	391.28538	3.89	1.03982	19.04	0.26947	22.11	0.277056
Cholic acid	C24H40O5		C00695	HRM${\text{S}}^{1}_{\text{a}}$, R_ta_	407.28030	4.04	1.27125	17.44	0.20141	20.21	0.272061
Dehydrocholic acid	C24H34O5		C13154	HRM${\text{S}}^{1}_{\text{a}}$, R_ta_	401.23335	3.77	0.93236	16.21	0.35086	18.79	0.544383
Chenodeoxycholic acid	C24H40O4		C02528	HRM${\text{S}}^{1}_{\text{a}}$, R_ta_	391.28538	4.03	1.00201	19.06	0.29212	22.11	0.277056

^a^See reference Sumner et al. (2014)

The non-polar bile acids that were retained on the RPLC column were not retained when using the pHILIC column alone (Fig. 3b, a, Supplementary Information). In contrast to the pHILIC, when the compounds were analysed using the RPLC/HILIC method, all of the bile acids were retained (Fig. 3c, Supplementary Information). The RPLC/HILIC method showed good retention time reproducibility, with RSD values for each compound under 5 % (Table 1).

### Beer sample

Beer components, suggested from previous beer metabolome studies, were searched for using their m/z values^1^ (Farag et al. 2012) (their structures can be seen in Fig. 4 in the Supplementary Information).

The small polar metabolites (putatively identified as syringaldehyde, tyrosine, ethyl vanillin, succinic acid, phenylalanine) that were retained on the pHILIC column were not retained when using the RPLC column alone (Fig. 2a, b). In contrast to the RPLC, when the compounds were analysed using the RPLC/HILIC method, all of the selected polar beer metabolites were retained and separated (Fig. 2c). The RPLC/HILIC method showed good retention time reproducibility, with RSD values for each compound under 5 % (Table 2, Supplementary Information).

The larger non-polar metabolites (putatively identified as ad-/humulone, iso-/xanthohumol and cohumulone) that were retained on the RPLC column were not retained when using the pHILIC column alone (Fig. 2b, a). In contrast to the pHILIC, when the compounds were analysed using the RPLC/HILIC method, all of the selected non-polar beer metabolites were retained and separated (Fig. 2c). As observed with the polar metabolites, all the non-polar metabolites had RSD values under 5 % (Table 2, Supplementary Information).

Serial coupling of RPLC and pHILIC was developed to separate polar and nonpolar compounds in a single HPLC–MS run. The first column selected was the RP column, followed by the pHILIC column. Two independent UPHLC pumps were incorporated into the system to allow independent gradient control of the two columns, so that different chromatography conditions could be applied to each phase. RPLC starting conditions were set to a low organic concentration and high aqueous concentration, whereas HILIC starting conditions were set to a high organic solvent concentration. The sample in low organic solvent concentration was injected onto the first column at a flow rate of 65 µL/min. Using this setup, non-polar metabolites were retained by the C18/RP stationary phase. Polar and ionic metabolites that were not retained on the RP column entered the T-piece where the second pump, which was running at a flow rate of 350 µL/min, and were then captured by the HILIC stationary phase. The dilution effect results in the eluent from the RP column failing to significantly affect the strength of the solvent entering the HILIC column, and gradients were then applied simultaneously to separate analytes on both columns.

The method described in this study was an adaptation of the methods described by Louw et al. (2008) and Greco et al. (2013). Louw ran a C18 2 mm ID column at a flow rate of 200 µL/min and a HILIC 4 mm ID column at a flow rate of 1.4 mL/min. Greco suggested that the high flow rates used may affect the sensitivity of MS detection and so used a longer C18 column, with a 3 mm ID, and a shorter HILIC column, with a 2 mm ID. They also ran at lower flow rates of 50–100 and 400 µL/min respectively. In the experiments described in this paper a 1 mm ID C18 and a 4 mm ID pHILIC were run at a flow rate of 65 and 350 µL/min, respectively.

Unlike Louw and Greco, the samples in this study were prepared in high aqueous solutions, the optimum conditions for initial injection onto the RP column.

Ion pairing reagents were added to the RP mobile phases in both the Louw and Greco papers (and to the HILIC mobile phase in Louw et al.). In the experiments described herein, ion pairing reagents were not introduced into the RP buffers. Ion pairing reagents are usually introduced into a RP separation to mask the ionic or polar groups of compounds to encourage binding to the RP stationary phase. In this experiment this was not desirable as the HILIC column after the RP column captured and separated the ionic and polar compounds in the sample. 20 mM ammonium bicarbonate was added to the HILIC mobile phase to encourage retention of the polar and ionic compounds by increasing the pH to 9.

The high dilution between the columns, achieved by the difference in flow rates, allows each of the methods to be optimised independently to suit sample requirements. The ability to inject the sample in low organic solvents onto the RPLC column and high organic solvents onto the HILIC column has allowed two very different column chemistries to be successfully coupled together.

The standards and the selected polar and non-polar metabolites extracted from beer displayed better retention and separation using the RPLC/HILIC method compared to the individual runs. All metabolites exhibited good peak shapes and reproducibility.

The results from this study highlight the advantages of using the RPLC/HILIC method for the separation and detection of both polar and non-polar metabolites, within a complex sample, in a single run. This method could improve analytical efficiency by halving the time required for analysis and removing the need for complicated system set-ups or sample preparations. We believe this method will have broad application in the field of metabolomics, as it allows significantly increased separation capacity for both polar and non-polar compounds without compromising on run time.

## Electronic supplementary material

Below is the link to the electronic supplementary material.

Supplementary material 1 (DOCX 1253 kb)

## Footnotes

1 Ullucci, P. A., et al. Chalconoids and Bitter Acids in Beer by HPLC with UV and Electrochemical Detection, Application Note 1020, Thermo Fisher.
