# Supplementary material for: Serially coupling hydrophobic interaction and reversed-phase chromatography with simultaneous gradients provides greater coverage of the metabolome
Source: Metabolomics. 2015 Jan 11;11(5):1465–70. doi: 10.1007/s11306-014-0770-7 (PMC4559102; doi:10.1007/s11306-014-0770-7)
Supplement: Supplementary file 1 — Supplementary material 1 (DOCX 1253 kb) [file 11306_2014_770_MOESM1_ESM.docx]

**Supplementary information**

**

**Fig 1** Structures of the test compounds

**Table 1.** Chromatographic conditions for the combined RPLC/pHILIC method

|  | Pump 1 | | |  |  | Pump 2 | | |
| --- | --- | --- | --- | --- | --- | --- | --- | --- |
| Solvent A | H_2_O | | |  | Solvent A | H_2_O + 20 mM ammonium carbonate | | |
| Solvent B | ACN | | |  | Solvent B | ACN | | |
| Column | Thermo Hypersil GOLD (100 x 1.0 mm, 1.9 µm) | | |  | Column | SeQuant® ZIC®-pHILIC (150 x 4.6 mm, 5 µm) | | |
|  |  |  |  |  |  |  |  |  |
| Gradient | Time (min) | B% | Flow (mL/min) |  | Gradient | Time (min) | B% | Flow (mL/min) |
|  | 0 | 5 | 0.065 |  |  | 0 | 90 | 0.350 |
|  | 2 | 5 | 0.065 |  |  | 5 | 90 | 0.350 |
|  | 17 | 95 | 0.065 |  |  | 20 | 20 | 0.350 |
|  | 22 | 95 | 0.065 |  |  | 20.1 | 5 | 0.350 |
|  | 22.1 | 5 | 0.065 |  |  | 25 | 5 | 0.350 |
|  |  |  | 0.065 |  |  | 25.1 | 90 | 0.350 |
|  | 37.5 | 5 | 0.065 |  |  | 37.5 | 90 | 0.350 |

**Fig 2** Extracted ion chromatograms (EICs) of selected organic acids using A): HILIC, B): RPLC and C): RPLC/HILIC separation using the same dual gradient. Injection volume 10 µL. Detection: ESI-FTMS, negative mode. Note: the second component of the double peak observed for the mass of fumarate on RPLC/HILIC EIC (C) is a consequence of water loss from malate.


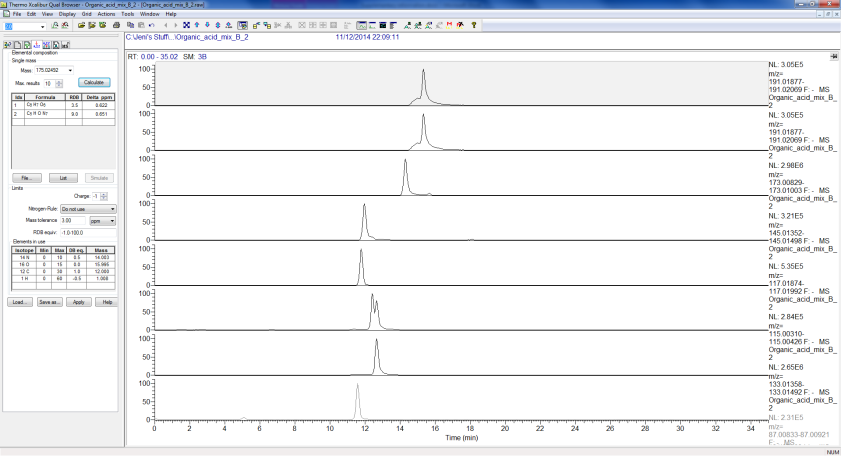

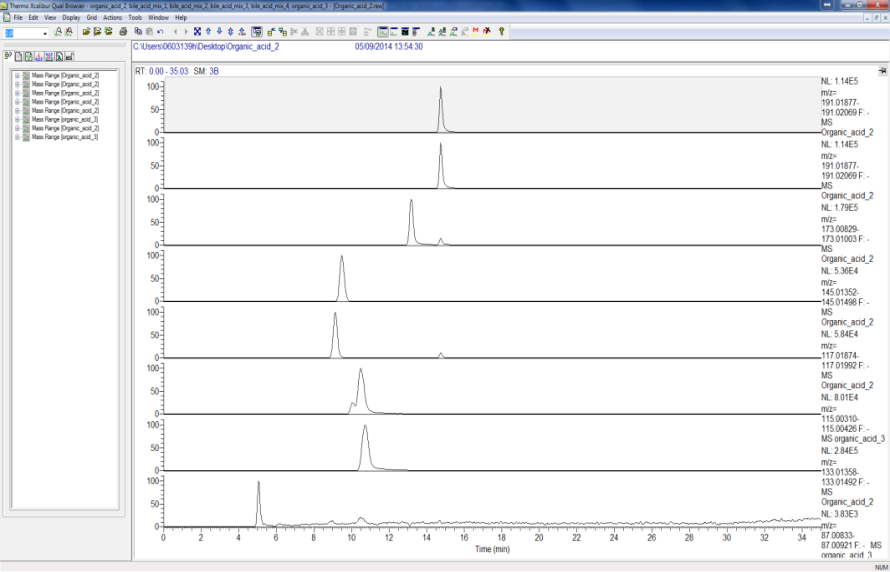

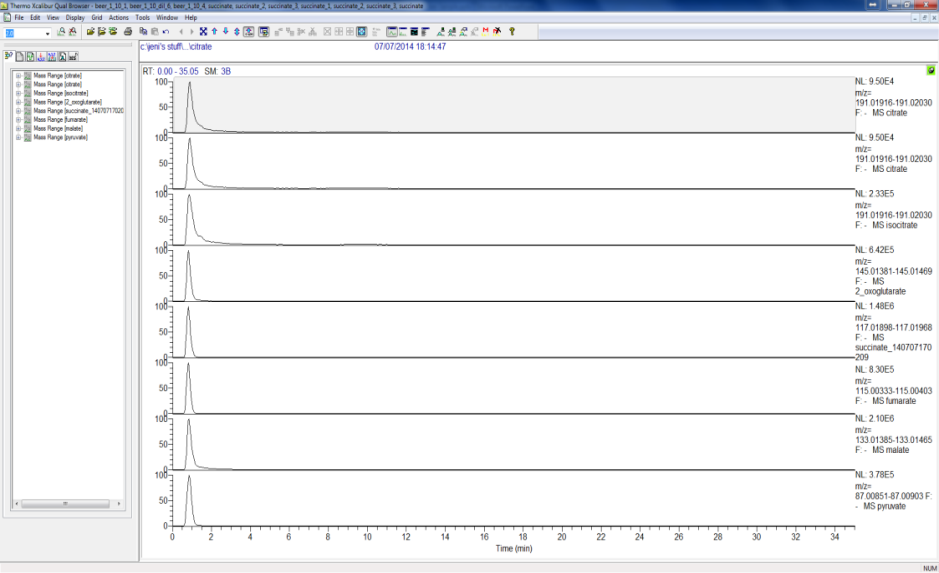


**HILIC (A)**

**RPLC/HILIC (C)**

Isocitric acid

cis-Aconitic acid

2-Oxoglutaric acid

Succinic acid

Fumaric acid

Malic acid

Pyruvic acid

**RPLC (B)**

pyruvate

fumarate

fumarate


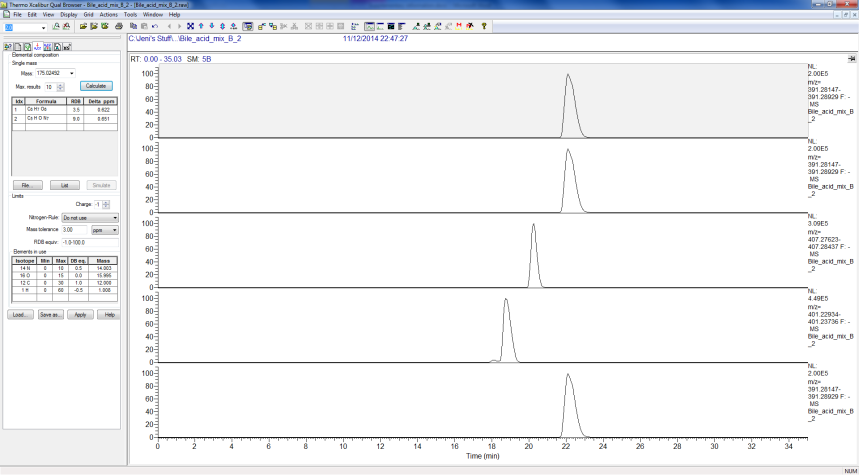

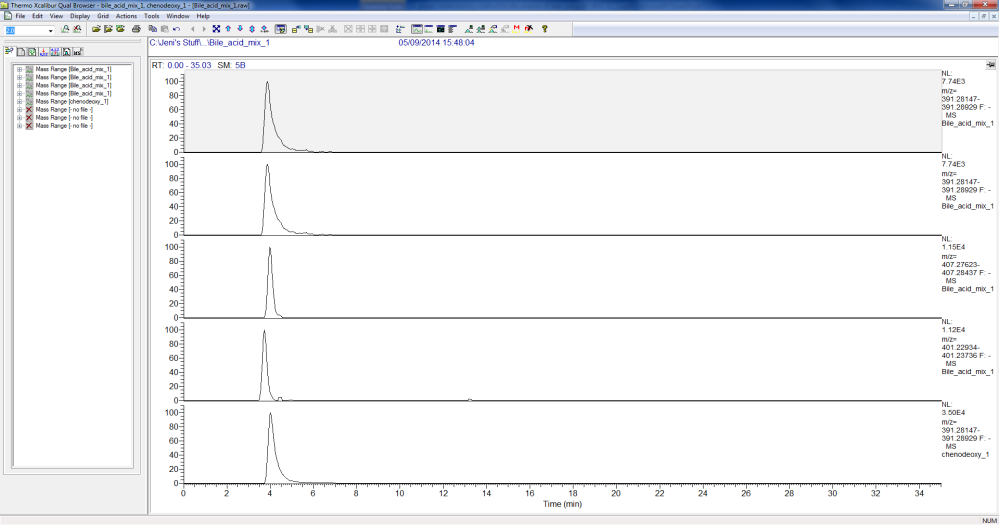

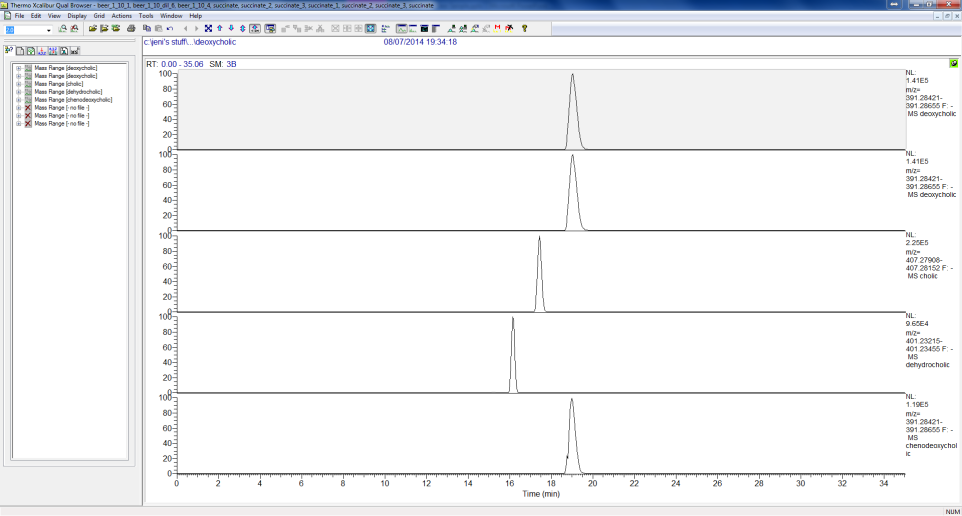


**RPLC (B)**

**RPLC/HILIC (C)**

**HILIC (A)**

Chenodeoxycholic acid

Dehydrocholic acid

Cholic acid

Deoxycholic acid

**Fig 3** EICs of selected bile acids using A): HILIC, B): RPLC and C): RPLC/HILIC separation using the same dual gradient. Injection volume 10 µL. Detection: ESI-FTMS, negative mode.

**Fig 4** Structures of putative beer metabolites

**Table 2**. Average retention times (RT) and RSDs of selected beer metabolites

|  |  |  |  |  | **HILIC** | | **RPLC** | | **RPLC/HILIC** | |
| --- | --- | --- | --- | --- | --- | --- | --- | --- | --- | --- |
| **m/z** | **EF** | **Putative Metabolite** | **Metabolite ID code^**^** | **KEGG ID** | **Average RT(mins)** | **%RSD** | **Average RT(mins)** | **%RSD** | **Average RT(mins)** | **%RSD** |
| 361.20205 | C21H30O5 | Ad-/Humulone | HRMS^1^ | -/C10695 | 4.25 | 6.64507 | 16.69 | 0.67358 | 19.02 | 0.18761 |
| 353.13945 | C21H22O5 | Iso-/Xanthohumol | HRMS^1^ | -/C16417 | 18.11 | 2.03559 | 19.18 | 0.29652 | 23.66 | 0.28152 |
| 347.18648 | C20H28O5 | Cohumulone | HRMS^1^ | N/A | 3.98 | 1.59254 | 16.20 | 0.29603 | 18.56 | 0.10422 |
| 181.05063 | C9H10O4 | Syringaldehyde | HRMS^1^ | N/A | 5.51 | 0.36298 | 1.27 | 0.00000 | 5.76 | 0.86212 |
| 182.08101 | C9H11NO3 | Tyrosine* | HRMS^1^MS^2^_PL_ | C00082 | 11.04 | 0.46977 | 1.58 | 1.16190 | 11.46 | 0.39842 |
| 165.05572 | C9H10O3 | Ethyl vanillin | HRMS^1^ | D01086 | 4.39 | 0.00000 | 1.59 | 1.58610 | 4.57 | 0.90847 |
| 117.01973 | C4H6O4 | Succinic acid | HRMS^1^MS^2^_PL_ | C00042 | 8.78 | 0.87022 | 1.12 | 6.83967 | 12.03 | 0.55094 |
| 166.08625 | C9H11NO2 | Phenylalanine* | HRMS^1^MS^2^_PL_ | C00079 | 8.02 | 0.07196 | 1.78 | 0.85977 | 8.44 | 0.35629 |

*Detected in positive mode, ** Refers to alphanumeric coding scheme from (Sumner *et al*. 2014)
